# Spen modulates lipid droplet content in adult *Drosophila* glial cells and protects against paraquat toxicity

**DOI:** 10.1038/s41598-020-76891-9

**Published:** 2020-11-18

**Authors:** Victor Girard, Valérie Goubard, Matthieu Querenet, Laurent Seugnet, Laurent Pays, Serge Nataf, Eloïse Dufourd, David Cluet, Bertrand Mollereau, Nathalie Davoust

**Affiliations:** 1 Laboratory of Biology and Modelling of the Cell, UMR5239 CNRS, INSERM U 1210, ENS de Lyon, UMS 344 Biosciences Lyon Gerland, Université de Lyon, Lyon, France; 2 CarMeN Laboratory, INSERM UMR-1060, INRA U1235, INSA of Lyon, Charles Merieux Medical School, Université Claude Bernard Lyon1, Université de Lyon, Lyon, France; 3 Banque de Tissus et de Cellules des Hospices Civils de Lyon, Hôpital Edouard Herriot, Lyon, France; 4 Centre de Recherche en Neurosciences de Lyon, UMR5292, INSERM U1028, Equipe Physiologie Intégrée du Système d’éveil, Université Claude Bernard Lyon1, Lyon, France; 5 Institut Universitaire de France, Paris, France

**Keywords:** Parkinson's disease, Astrocyte, Disease model

## Abstract

Glial cells are early sensors of neuronal injury and can store lipids in lipid droplets under oxidative stress conditions. Here, we investigated the functions of the RNA-binding protein, *SPEN/SHARP,* in the context of Parkinson’s disease (PD). Using a data-mining approach, we found that *SPEN/SHARP* is one of many astrocyte-expressed genes that are significantly differentially expressed in the *substantia nigra* of PD patients compared with control subjects. Interestingly, the differentially expressed genes are enriched in lipid metabolism-associated genes. In a *Drosophila* model of PD, we observed that flies carrying a loss-of-function allele of the ortholog *split-ends* (*spen*) or with glial cell-specific, but not neuronal-specific, *spen* knockdown were more sensitive to paraquat intoxication, indicating a protective role for Spen in glial cells. We also found that Spen is a positive regulator of Notch signaling in adult *Drosophila* glial cells. Moreover, Spen was required to limit abnormal accumulation of lipid droplets in glial cells in a manner independent of its regulation of Notch signaling. Taken together, our results demonstrate that Spen regulates lipid metabolism and storage in glial cells and contributes to glial cell-mediated neuroprotection.

## Introduction

Parkinson’s disease (PD) is a neurodegenerative disorder characterized by the selective loss of dopaminergic neurons in the *substantia nigra pars compacta* (SN). Although the etiology of PD remains unclear, environmental factors combined with a permissive genetic background are thought to contribute. Epidemiologic studies have shown that chronic exposure to pesticides is one environmental factor involved in the development of PD^1^. Several models of PD have been developed in *Drosophila*, among which the paraquat-induced model reproduces several important pathophysiological features of the disease, thereby enabling causal links between chronic pesticide intoxication and PD to be investigated in detail. In particular, oxidative stress and reactive oxygen species (ROS) production in the brain of paraquat-intoxicated flies has been shown to induce the degeneration of dopaminergic neurons, leading to severe motor disability and premature death^2–4^.

In many species including *Drosophila* and humans, non-neuronal glial cells are early sensors of central nervous system injury^5^. Glial cells such as microglia and astrocytes respond to neuronal damage by changing their morphology and proliferation and activating specific transcriptomic programs. We and others recently demonstrated that intracytoplasmic accumulation of lipid droplets (LDs) is a hallmark of the glial cell response to stress^6–10^. Recent work has shown that the regulation of lipid metabolism and formation of LDs requires the RNA-binding protein Split-ends (*spen)*, the *Drosophila* ortholog of *SPEN/SHARP,* in *Drosophila* adipose tissue^11,12^. Spen also regulates midline glia specification and survival of glial cells in the *Drosophila* embryo^13^ and pupal retina^14^, respectively. However, the role of Spen in the regulation of lipid metabolism in glial cells is unknown. Spen proteins contain two conserved functional domains: an RNA Recognition Motif (RMM) and a Spen Paralog and Ortholog C-terminal (SPOC) domain^13,15–17^*,* which mediate its biological effects through transcription, RNA silencing, RNA splicing, and direct interactions with chromatin^18–23^.

*SPEN/SHARP* can act as both a negative and positive regulator of the Notch signaling pathway, which plays a major role in cell fate specification in many species^24–27^. A recent study showed that SPEN acts as co-repressor of the transcription of genes responsive to Notch signaling^25^ by binding to the recombinant binding protein J-kappa (RBP-Jκ) during *Drosophila* eye development^26^*.* Conversely, SPEN can act as a positive regulator of Notch signaling by recruiting the lysine methyl transferase 2D (KMT2D) co-activator complex to Notch target genes^27^. Spen has also been shown to promote Notch receptor activation by regulating trafficking of the Notch ligand Delta in intestinal stem cells of adult flies^28^.

Given that Spen promotes survival in glial cells of the developing *Drosophila*, we hypothesized that Spen expression in glial cells could confer neuroprotection in a model of PD. In the present study, we investigated the role of Spen as a potential regulator of lipid metabolism and Notch signaling in adult *Drosophila* glial cells, and determined the possible involvement of *spen* in the paraquat intoxication model of PD.

## Results

### *SPEN*, the human ortholog of *Drosophila spen*, is upregulated in the *substantia nigra pars compacta* of PD patients

*SPEN* has been reported to be expressed by human astrocytes^29^, but whether its expression is differentially regulated in the brains of patients with PD is unknown. To address this, we exploited the findings of a recent meta-analysis of microarray datasets obtained from the SN of PD patients compared with control subjects^30^. From the list of differentially expressed genes (data Supplement 1), we performed a tissue enrichment analysis using the TargetMine webtool^31^. This analysis identified 197 genes that were both upregulated in the SN of PD patients and expressed in astrocytes; one which was SPEN. SPEN is known to be expressed by both astrocytes and neurons in the normal human brain^29^ (data Supplement 2); however, it is not clear whether the upregulation of SPEN in the brains of PD patients occurs in glia or neurons. We also identified 469 genes that were both downregulated in the SN of PD patients compared with control subjects and expressed in astrocytes (data Supplement 2). Interestingly, we detected significant enrichment of lipid metabolism-associated genes, as determined using the BioPlanet pathway enrichment tool^32^, in the gene set downregulated, but not upregulated, in the SN of PD patients (data Supplement S3, S4, Table 1). Taken together, these analyses indicate that *SPEN* expression is upregulated in astrocytes and/or neurons of PD patients compared with normal subjects, which prompted us to investigate its function in the *Drosophila* paraquat model of PD.

**Table 1:** Genes involved in lipid metabolism that are downregulated in the *substantia nigra pars compacta* of PD patients compared with control subjects.

Pathway	Human genes	Adjusted P-value
Phospholipid metabolism	*ARF3, ASAH1, CERS6, CERK, PITPNB, PIK3R3, OCRL, MTMR4, PLD3, AGPAT4, INPP4A, PTDSS1, COL4A3BP, STS, SYNJ1, VAPB, OSBP, PI4KA, PIP5K1B, GPD1L, PI4K2A, CDS2*	0.0003
Lipid and lipoprotein metabolism	*ARF3, IDI1, ASAH1, ALAS1, CERK, PITPNB, PIK3R3, HMGCR, MTMR4, PLD3, AGPAT4, PTDSS1, COL4A3BP, STS, OXCT1, OSBP, PIP5K1B, PRKACB, CERS6, HMGCS1, SRD5A1, OCRL, MED7, INPP4A, ACLY, SYNJ1, VAPB, AGPS, PI4KA, GPD1L, PI4K2A, CDS2*	0.0180
Sphingolipid metabolism	*COL4A3BP, ASAH1, STS, CERS6, CERK, VAPB, OSBP, B4GALT6*	0.0488

### Glia-specific overexpression of *spen* protects *Drosophila* from paraquat-induced neurotoxicity

**Figure 1:**
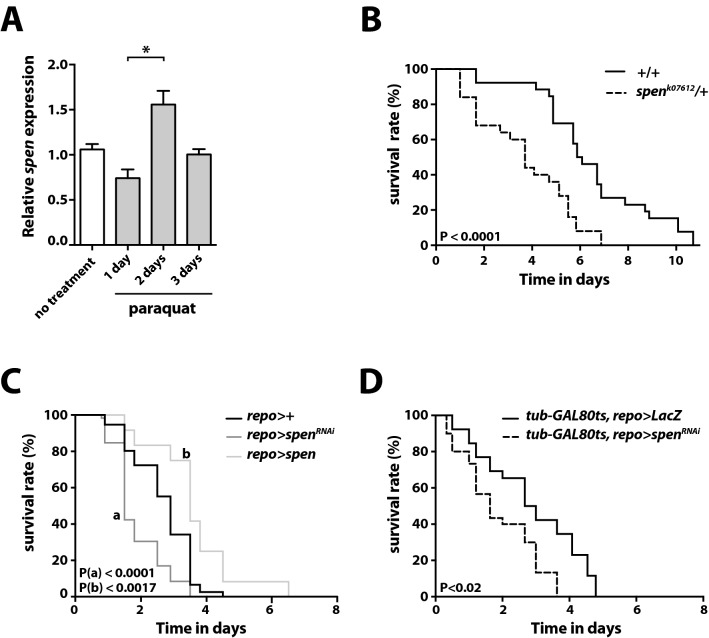
Glia-specific *spen* expression protects *Drosophila* against paraquat-induced lethality. (**A**) RT-qPCR analysis of *spen* mRNA expression in the heads of 3-day-old male *Drosophila* at the indicated times after feeding with a standard sucrose solution lacking (no treatment) or containing 10 mM paraquat for the indicated times (4 independent experiments). Results are expressed relative to the no-treatment condition. *P < 0.03 by nonparametric Mann–Whitney test. (**B**) Survival curves of wild-type (*w*^*1118*^) and *spen* loss-of-function heterozygous mutant (*spen*^*k07612*^*/* +) adult male flies fed with 10 mM paraquat*.* The curves represent one experiment with N = 16–20 flies per genotype, representative of three independent experiments. P < 0.0001 by the log-rank Mantel–Cox test. (**C**) Survival curves of adult male control flies (*repo-GAL4/* +) or flies with glial cell-specific *spen* knockdown (*UAS-spen*^*RNAi*^*/* +*;repo-GAL4/* +) or *spen* overexpression (*UAS-spen/* +*;repo-GAL4/* +) fed with 10 mM paraquat. The curves represent one experiment with N = 20 flies per genotype, representative of three independent experiments. (a) P < 0.0001 for control vs *repo* > *spen*^*RNAi*^, (b) P < 0.0017 for control vs *repo* > *spen* by the log-rank Mantel–Cox test. (**D**) Survival curves of adult male flies with expression of *LacZ* (control) or *spen*^*RNAi*^ restricted to adult glial cells using the TARGET system^45^. Flies were fed 10 mM paraquat for the indicated times. The curves represent one experiment with N = 16–20 flies per genotype, representative of three independent experiments. P < 0.02 by the log-rank Mantel–Cox test.

We first investigated whether *spen* mRNA levels in the brain of adult *Drosophila* were altered under conditions of paraquat-induced toxicity. RT-qPCR revealed a significant upregulation of *spen* expression in the brains of paraquat-treated flies compared with control flies (Fig. 1A). To obtain insights on the potential function of Spen upregulation, we examined the survival of flies heterozygous for *spen* loss-of-function mutations^17^ (*spen*^*k07612*^*/*+ and *spen*^*03350*^*/*+) after paraquat intoxication. Both of the *spen* heterozygous mutant lines exhibited a higher sensitivity to paraquat compared with control flies (Fig. 1B, Supplemental Fig. S1), suggesting that Spen protects against paraquat-induced neurotoxicity. As previously reported^33^, *spen* expression, as revealed by P-lacW inserts, was detectable in both neurons and glial cells of *Drosophila* adult brain (Supplemental Fig. S2). To determine whether the neuroprotective function of *spen* results from its expression in glial or neuronal cells, we generated flies in which *spen* was selectively knocked down in either cell type using a pan-glial (*repo*) or pan-neuronal (*elav*) driver. We found that downregulation of *spen* in glial cells, but not neuronal cells, increased the sensitivity of male flies to paraquat (Fig. 1C, Supplemental Fig. S3). Knockdown of *spen* in adult glia using an alternative genetic method, the temperature-sensitive TARGET system^45^, had the same effect of increasing *Drosophila* sensitivity to paraquat (Fig. 1D). Conversely, glia-specific overexpression of *spen* protected against paraquat toxicity (Fig. 1C). Collectively, these results show that Spen expression in glial cells protects *Drosophila* from paraquat-induced toxicity.

### Spen regulates the Notch signaling pathway in adult Drosophila glial cells

**Figure 2:**
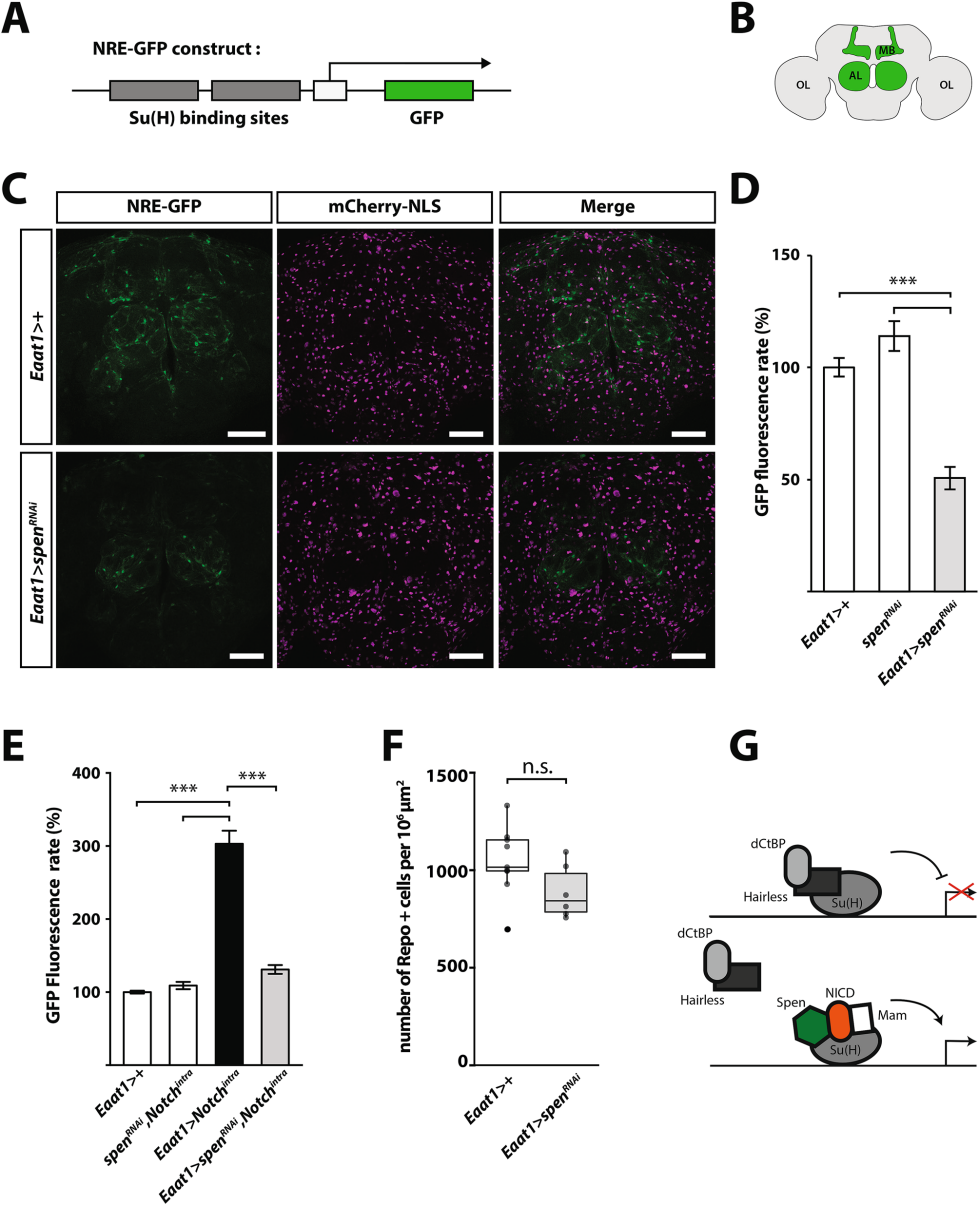
Spen regulates Notch signaling in *Drosophila* adult glial cells. (**A**) Schematic of the Notch reporter construct *NRE-GFP* used to monitor Notch activity in *Drosophila* brain. The *EGFP* coding sequence (green) is under the control of a promoter containing multiple Su(H) binding sites (dark gray). (**B**) Simplified schematic of *Drosophila* adult brain, showing mushroom bodies (MB) and antennal lobes (AL), which are known to have high Notch activity (green). OL, optic lobes. (**C**) Immunofluorescence confocal micrographs of whole-mount brains from control flies (*Eaat1* glial driver alone: *Eaat1-GAL4/* +*;NRE-GFP/* +) or flies with glia-specific *spen*^*RNAi*^ (*Eaat1-GAL4/UAS-spen*^*RNAi*^*;NRE-GFP/* +). Flies also harbored the nuclear marker mCherry-NLS expressed under the control of the *Eaat1* driver (magenta). GFP fluorescence was detected in MB and AL lobes in close proximity with *Eaat1* + glial cells. The NRE-GFP signal is reduced in *Eaat1* > *spen*^*RNAi*^ (*Eaat1-GAL4/UAS-spen*^*RNAi*^*; NRE-GFP/* +) flies compared with controls. Scale bar, 50 μm. (**D**) Quantification of NRE-GFP fluorescence in flies with driver alone *Eaat1* >  + (*Eaat1-GAL4/* +*;NRE-GFP/* +)*, UAS*-*spen*^*RNAi*^ alone (*UAS-spen*^*RNAi*^*/* +*;NRE-GFP/* +), or *spen*^*RNAi*^* Eaat1* > *spen*^*RNAi*^ (*Eaat1-GAL4/UAS-spen*^*RNAi*^*; NRE-GFP/* +). N = 18 flies per genotype from 3 independent experiments. (**E**) NRE-GFP fluorescence was quantified in *Eaat1* >  + (*Eaat1-GAL4/* +*;NRE-GFP/* +), *UAS-spen*^*RNAi*^ (*UAS-spen*^*RNAi*^*, UAS-Notch*^*intra*^*;NRE-GFP/*)*,* or Notch intracellular domain-overexpressing (*Eaat1-GAL4/UAS-Notch*^*intra*^*; NRE-GFP/* +) flies alone or together with *spen*^*RNAi*^ (*Eaat1-GAL4/ UAS-spen*^*RNAi*^*, UAS-Notch*^*intra*^*;NRE-GFP/* +)*.* N = 18 flies per genotype from 3 independent experiments. P < 0.001 by the nonparametric Kruskal–Wallis test. (**F**) Density of Repo-positive glial cells in control (*Eaat1-GAL4/* +) or *spen*^*RNAi*^ flies (*Eaat1-GAL4/UAS-spen*^*RNAi*^). Boxes show the median and upper, and lower quartiles, and the whiskers represent 1.5 times the interquartile range. Repo-positive glial cells were quantified over 12 confocal slices across one 12 µm-thick brain section from each of *Drosophila* brains. Circles represent individual data points. (**G**) Simplified proposed scheme showing the Notch activator complex in adult glial cells, which includes the Notch intracellular domain (NICD), Suppressor of hairless (Su(H)), mastermind (Mam), and Spen. The Notch repressor complex includes Su(H), Hairless, and dCtBP (*Drosophila* C-terminal Binding Protein). Adapted from^38^.

Because Spen/SPEN has been shown to positively or negatively regulate Notch signaling pathway, depending on the molecular context and cell type^25,27,28^, we next determined how Spen regulates Notch pathway in adult glial cells. To this end, we knocked down *spen* expression using an *Eaat1* (excitatory amino acid transporter 1) driver, which is mainly expressed in astrocyte-like glial cells^34,35^, and monitored Notch pathway activation using a reporter transgene, Notch Responsive Element (*NRE)-GFP*. This transgene carries a minimal promoter containing Su(H)-DNA binding sites upstream of the *EGFP* coding sequence^36^ (Fig. 2A). As previously reported, basal expression of *NRE-GFP,* reflecting basal activity of the Notch pathway, was observed in glial cells of control flies (*Eaat1* > +), particularly in the antennal lobe^37^ (Fig. 2B,C, Supplemental Fig. S4) and the dorsal part of the central brain. Interestingly, however, basal activity of Notch signaling was strongly diminished in flies with glia-specific expression of *spen*^*RNAi*^ (Fig. 2C,D). These data indicate that Spen is required for basal Notch activity in glial cells in the adult *Drosophila* brain. To extend these observations, we performed an epistasis experiment between the Notch intracellular domain (Notch^intra^), an activator of the Notch pathway, and *spen*^*RNAi*^ in Eaat1+ cells. As expected, Notch^intra^ expression resulted in a strong and uniform induction of *NRE-GFP* reporter expression; however, *spen* knockdown significantly reduced the induction of NRE-GFP by Notch^intra^, indicating that Spen is necessary to maintain Su(H)-dependent Notch signaling in adult glia (Fig. 2D,E). The induction of NRE-GFP was not associated with a change in glial cell survival rate, as assessed by quantification of Repo-positive cells in control and *spen* knockdown flies (Fig. 2F). Collectively, these results show that Spen acts as a positive regulator of Notch signaling in adult *Drosophila* glial cells (Fig. 2G).

### Spen regulates the number, size, and localization of lipid droplets in glial cells

**Figure 3:**
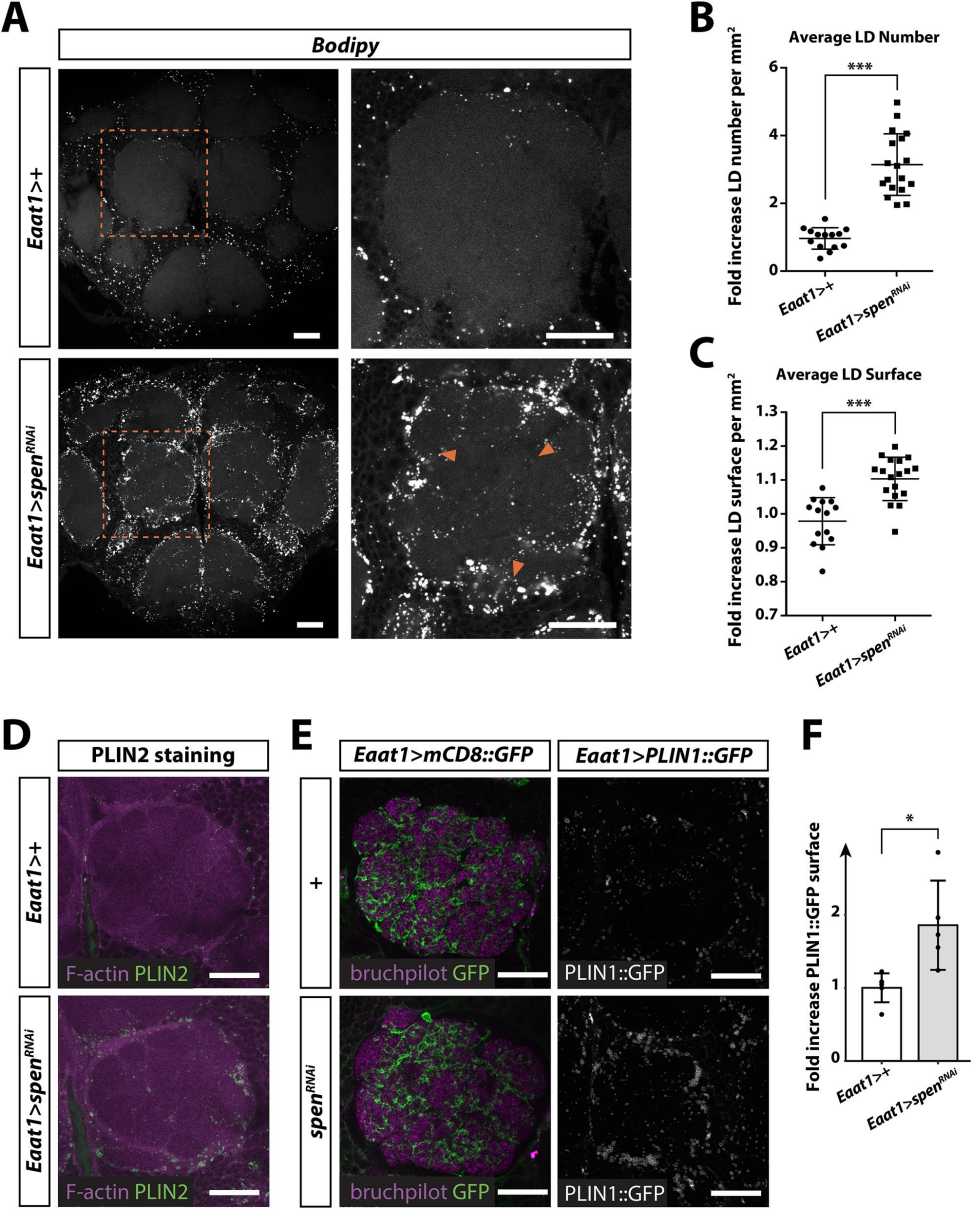
Spen regulates lipid droplet number, size, and localization in *Drosophila* glial cells. (**A**) Fluorescence micrographs showing lipid droplets (LDs; white dots, BODIPY 493/503) in whole-mount brains of control flies *Eaat1* >  + (*Eaat1-GAL4/* + ) or flies expressing *spen*^*RNAi*^ in glial cells *Eaat1* > *spen*^*RNAi*^ (*Eaat1-GAL4/UAS-spen*^*RNAi*^). Right panels show magnifications of the boxes outlined in the left panels. Orange arrowheads indicate LDs accumulated in the neuropil area of the antennal lobe in *spen*^*RNAi*^ flies. Scale bar, 25 µm. (**B**,**C**) Quantification of LD number (**B**) and LD surface (**C**) in *Eaat1* > *spen*^*RNAi*^ flies relative to the control flies *Eaat1* >  + (*Eaat1-GAL4/* +). N = 14–18 brains per genotype from 3 independent experiments. Circles represent individual data points. LDs were quantified using an automated ImageJ plugin (see “Materials and methods” section). P < 0.0001 by unpaired Student’s t test. (**D**) Fluorescence micrographs showing PLIN2 antibody staining (green) in whole-mount brains from control flies *Eaat1* >  + (*Eaat1-GAL4/* +) and *spen*^*RNAi*^ flies (*Eaat1-GAL4/UAS-spen*^*RNAi*^). Brains were counterstained with phalloidin-rhodamine to detect F-actin (magenta). (**E**) Fluorescence micrographs of the antennal lobe of: (left panel) flies expressing the membrane reporter *mCD8::GFP* alone or in conjunction with *spen*^*RNAi*^ under the control of the *Eaat1-GAL4* driver to label astrocyte-like glial processes infiltrating the antennal lobe, and (right panel) flies expressing the lipid droplet reporter *UAS-PLIN1::GFP (PLIN1::GFP)* alone or in conjunction with *spen*^*RNAi*^ under the control of *Eaat1-GAL4.* Scale bar, 25 µm. (**F**) Quantification of PLIN1::GFP-positive staining in *Eaat1* > *spen*^*RNAi*^ flies relative to control flies *Eaat1* >  + (*Eaat1-GAL4/* +) flies. N = 5 brains per genotype. Circles represent individual data points. *P < 0.05 by unpaired Student’s t test.

*Drosophila* glial cells have been shown to accumulate LDs under conditions of oxidative stress^6,7^, and Spen is known to control lipid metabolism in the fat body of *Drosophila* larvae^11^. We thus investigated whether Spen expression affects LD expansion and/or accumulation in *Drosophila* glial cells. To this end, we developed an ImageJ^39^ macro to quantify fluorescence from BODIPY staining of LDs in serial confocal sections of adult *Drosophila* brain. Quantification was based on an automated detection of BODIPY-positive particles that were distinguished from non-specific fluorescence by successive iterations. This new method allows not only discrimination of LDs from doublet foci on successive confocal stacks but also evaluation of the density, size, and circularity of LDs (see “Materials and methods” section for details). As visualized in the fluorescence micrographs (Fig. 3A) and quantified in the antennal lobe neuropil (Fig. 3B,C), *spen* knockdown in glial cells induced a significant increase in LD number and size. A similar accumulation of LDs was seen when *spen*^*RNAi*^ was expressed using the pan-glial driver *repo-GAL4*, but not the pan-neuronal driver *elav-GAL4* (Supplemental Fig. S5). The accumulation of LDs induced by glia-specific knockdown of *spen* was also detected when LDs were visualized by staining of whole-mount brains for PLIN2^40,41^, a peridroplet protein anchored to the phospholipid monolayer surrounding LDs (Fig. 3D). In addition to the immunostaining approach, we expressed the *UAS-PLIN1::GFP* genetically encoded LD reporter^40,41^ under the control of the *Eaat1-GAL4* driver and confirmed that glia-specific *spen* knockdown induced accumulation of LDs in the brain of adult flies (Fig. 3E,F). Interestingly, inhibition of the canonical Notch pathway in glia by knockdown of *Su(H)* did not affect the number or size of LDs (Supplemental Fig. S6). Collectively these results suggest that LD accumulation in flies with *spen* knockdown occurs specifically in glial cells and is not due to the inhibition of canonical Notch signaling.

## Discussion

Several studies have reported that dysregulation of lipids, including LDs, is a component of the glial cell response to stress during neurodegeneration^6,8–10^, including that associated with PD^42^. Here, we identified *SPEN/SHARP* as an astrocyte-expressed gene that is significantly overexpressed in the SN of PD patients compared with control subjects. In *Drosophila*, *spen* expression in glial cells mediates the resistance of *Drosophila* to paraquat treatment. We also show that Spen is a positive regulator of Notch signalling in adult glial cells. Finally, we found that glia-specific expression of *spen* regulates LD accumulation in glial cells, in a manner independent of its regulation of Notch signaling. Collectively, our results suggest that the regulation of lipid metabolism by Spen contribute to the glia stress response*.*

*SPEN* was one of many astrocyte-expressed genes found to be significantly differentially expressed in the brains of PD patients compared with control subjects. Interestingly, the differentially expressed genes were enriched in genes involved in lipid metabolism, which was of particular interest given the previously reported role of Spen as a regulator of lipid storage in adipocyte-like cells in *Drosophila*. Indeed, Spen was identified in two independent screens as a modulator of fat content in *Drosophila* larvae and adults^43,44^. A more recent study showed that *spen* manipulation in adipocyte-like cells correlated with alterations in the expression of key metabolic enzymes, supporting a role for Spen in energy catabolism^11^. In the present study, we found that glia-specific silencing of *spen* also affected lipid metabolism, as reflected by an increase in the number and size of LDs in *Drosophila* glial cells. While the mechanism underlying this effect is unclear, it is possible that *spen* downregulation may lead to a decrease in LD degradation or an increase in expression of triacylglycerol biosynthesis genes. Further investigations will be required to determine if Spen acts as a positive or negative regulator of lipid metabolism in different physiological and pathological contexts.

Spen has been shown to function as a negative regulator of Notch signaling at the morphogenetic furrow during eye development in *Drosophila*^26^. Here, we showed that Spen positively regulates Notch signaling in adult *Drosophila* glial cells. Spen-dependent activation of the Notch pathway has also been observed in intestinal stem cells of adult flies^28^, substantiating the role of Spen as a positive regulator of Notch in adult tissues. Thus, Spen appears to differentially regulate Notch signaling in cell type-, context-, and developmental stage-specific manners. The ability of Spen to differentially affect Notch signaling may be mediated via recruitment of intermediate positive or negative regulatory factors, resulting in activation or repression of Notch responsive-genes expression, respectively.

Our results show that the accumulation of LDs induced by *spen* knockdown is independent of Notch/Su(H) signaling, as reflected by the lack of effect of glia-specific *Su(H)* knockdown on LD size or number. Rather, Spen may directly affect mRNA stability or splicing of lipid metabolism genes to promote gene expression independently of Notch^11^. Finally, it is possible that the LD accumulation may be due to increased oxidative stress in *spen*-mutant flies, as previously observed in flies with defective mitochondrial respiration or after exposure to hypoxia^6,7,9^. Our results showing that *spen*-mutant flies exhibited increased sensitivity to paraquat toxicity are consistent with a mechanism involving oxidative stress.

Our findings are also in accord with a role for glia-expressed Spen in lipid metabolism in the context of PD pathophysiology. Using a data-mining approach, we found that genes differentially expressed in the SN of PD patients are not only enriched in astrocyte-expressed genes (e.g., *SPEN/SHARP*) but also include a significant number of genes annotated with the Gene Ontology terms “phospholipid metabolism”, “lipid and lipoprotein metabolism”, and “sphingolipid metabolism”. These results point to a potential pathological role for lipid metabolism in PD, which is in accordance with a meta-analysis of genome-wide association studies of PD^46^. Among the lipid-related genes identified here to be differentially expressed in the SN of PD patients are several that could contribute to the regulation of LD formation and/or fate. For example, *AGPAT4* (1-acylglycerol-3-phosphate O-acyltransferase 4) is involved in the synthesis of precursors of TAGs, the major lipid component of LDs. Similarly, *SCD5* (stearoyl-CoA desaturase 5), a recently identified new target for PD treatment^47^, catalyzes free fatty acid desaturation and plays an important role in the early steps of LD formation. Finally, *Arf79F* and *schlank*, the *Drosophila* orthologs of two lipid-related genes found to be downregulated in the SN of PD patients, have been shown to control the homeostasis of LDs in *Drosophila*^48,49^.

Taken together, our data support a central role for *spen* expressed in glial cells in the control of lipid metabolism and resistance to paraquat-induced toxicity in *Drosophila*. Further studies on the function of Spen will contribute to our understanding of the involvement of lipid dyshomeostasis in neurodegeneration.

## Materials and methods

### Fly strains

Flies were maintained on standard yeast medium at 25 °C unless otherwise noted. Flies bearing the following mutations and transgenes were obtained from the Bloomington Drosophila Stock Center (Indiana University, IN, USA): *w*^*1118*^, *repo-GAL4* (BL7415), *UAS-mCherry-NLS* (BL38424), *tubulin-GAL80*^*ts*^ (BL7019), *UAS-mCD8::GFP* (BL32186), *NRE-EGFP1* (referred to as NRE-GFP, BL30728)*, UAS-Notch*^*intra*^ (BL52008), an *Eaat1-GAL4*^34^ (BL8849), *elav-GAL4* (BL458). *spen*^*k07612*^ and *spen*^*03350*^ P element insertions (*Drosophila* Genomics Resource Center [DGRC] #102574 and BL11295) were previously characterized as homozygous lethal *spen* loss-of-function mutations^17^ and were used here as heterozygotes. *UAS-PLIN1::GFP*^40^ was obtained from RP Kuhnlein (University of Graz, Austria). The EP line *spen-GS2279* (Kyoto DGRC Stock Center) was used to overexpress *spen,* and is referred to as *UAS-spen*. The *UAS-spen*^*RNAi*^ line was a gift from KM Cadigan^50^ and was previously characterized in studies of *Drosophila* retina development^14^. *spen* mutants and transgenic flies were outcrossed to a *w*^*1118*^ control stock. We used the temperature-sensitive TARGET system^45^ to restrict spen RNAi expression to adult glial cells. Briefly, flies carrying repo-GAL4, *tubulin-GAL80*^*ts*^, and *UAS-spen*^*RNAi*^ were raised at 18 °C to inhibit GAL4 activity and switched to 29 °C as adults to induce expression of spen^RNAi^.

### RT-qPCR

Total RNA was isolated from 25 to 35 *Drosophila* heads using RNeasy mini kits (Qiagen) and reverse transcribed with oligo(dT)15 primers and the ImProm-II Reverse Transcription System (Promega) according to manufacturers’ instructions. Quantitative PCR reactions were carried out on a StepOnePlus system (Applied Biosystems) using FastStart Universal SYBR Green Master (Roche Applied Science). Efficiency (E) of the primer sets was assessed using serial dilutions of cDNA preparations. Standard curves were generated to quantify mRNA abundance and PCR cycle numbers (Ct) for calculation of the relative mRNA expression level (Qr = E − Ct)^51^. Values were normalized to Rp49 mRNA levels. Primers for qPCR were: *spen* forward 5′-TTCGTTGTGGGATAGCAGCA-3′ and reverse 5′-CGTTCGAAGCTGTTTGTCG-3′ and *Rp49* forward 5′-ATCGTGAAGAAGCGCACCAAG-3′ and reverse 5′-ACCAGGAACTTCTTGAATCCG-3′.

### Immunostaining

*Drosophila* heads were removed and placed in a drop of fresh Hemolymph-Like 3 dissection buffer^52^ (HL3) supplemented with d-glucose (120 mM). The proboscis was removed and the cuticle was opened to access the brain, and the brains were dissected and fixed overnight at 4 °C in 1% paraformaldehyde (PFA) diluted in HL3 medium. Fixed brains were washed 3 times for 10 min each in phosphate-buffered saline (PBS) containing 0.5% Triton X-100 and 5 mg/ml bovine serum albumin, and then incubated for 1 h in the same buffer containing 4% normal goat serum to prevent non-specific antibody binding (blocking solution). The brains were then incubated overnight at 4 °C with the following primary antibodies diluted in blocking solution: mouse anti-Repo (1:400, Developmental Studies Hybridoma Bank, 8D12), rabbit anti-GFP (1:400, Invitrogen, A6455), and rabbit anti-PLIN2 (1:1000, a gift from RP Kuhnlein^40^). The samples were washed 3 times with PBS-T and incubated overnight at 4 °C with anti-mouse Alexa Fluor 647 (1:400, Invitrogen, A31571) or anti-rabbit Alexa Fluor 488 (1:400, Invitrogen A11008) secondary antibodies diluted in blocking solution. Samples were washed 3 times and then mounted in Vectashield mounting medium (AbCys) on a bridge slide to prevent tissue flattening. Samples were stored at − 20 °C until visualized.

For experiments with the NRE-GFP-expressing *Drosophila* line, the heads were removed and placed in a drop of fresh PBS. The brains were dissected and fixed in 4% PFA/PBS for 20 min at room temperature. The brains were then washed in PBS and mounted directly in Vectashield medium (AbCys). As controls for the NRE-GFP experiments, brains from *Drosophila* not expressing GFP were also processed to evaluate background fluorescence.

### Image processing

Images of whole-mount brains were acquired at the LYMIC-PLATIM Imaging and Microscopy Core Facility of SFR Biosciences (UMS3444, ENS de Lyon, France). For PLIN1::GFP, Repo, and BODIPY fluorescence, images were acquired on a Zeiss LSM800 confocal microscope and analyzed with the ImageJ^39^ software (see section below). For NRE-GFP fluorescence, images were acquired on a Leica epifluorescence microscope. In each experiment, the mean GFP fluorescence of control brains (*Eaat1-GAL4/*+) was used to normalize the results. The NRE reporter is a synthetic construct with three copies of SPS sites taken from the E(spl) regulatory region^36^. SPS (Su(H) paired sites) are binding sites for the Notch activity-dependent transcription factor Su(H).

### Automated image analysis

We developed an ImageJ macro (https://gitbio.ens-lyon.fr/dcluet/Lipid_Droplets) to identify fluorescent particles on confocal stacks using *Drosophila* brains stained with the lipid-binding dye BODIPY 493/503 (Molecular Probes, D-3922) or labeled with the glial cell-specific marker Repo. The program requires ImageJ^39^ v1.49 g or higher and is based on an iterative detection of the brightest particles followed by removal of “doublets” of the same particle over the stack. Briefly, the program first delineates the brain region of interest and then identifies particles within that region along the stack. The signal is intensified using the Gaussian blur function and the maximum entropy treatment. The “Max-Entropy” threshold method^53^ is then applied to detect the particles of interest. The detected particles are stored in a transient matrix and removed from the image. The next iteration is able to detect less bright particles. Finally, the program removes all doublets of the same particle along the z-dimension of the stack (keeping the largest candidate as the best) to enable optimal counting of labeled particles. The program can calculate multiple parameters, such as particle density, size, and circularity.

### Paraquat-induced PD model

Paraquat medium was prepared fresh shortly before each experiment. Paraquat (Sigma, 36541) was added at 10 or 20 mM to PBS (as indicated in the corresponding figure legends) containing 0.8% low-melting agarose (Sigma, A9414) and 10% sucrose (Sigma, S0389). Three-day-old male flies were fasted for 4 h on 0.8% agarose medium and then transferred to 10 or 20 mM paraquat-containing medium for 5–7 days for survival experiments or the indicated times for RT-qPCR analysis. At least three independent experiments were performed, each with n ≥ 20 flies per condition per experiment.

### Lipid droplet staining

Heads were removed from 6-day-old flies and placed in a drop of fresh HL3 dissection buffer supplemented with d-glucose (120 mM). Brains were dissected and fixed overnight at 4 °C in 1% PFA/HL3 medium. Fixed brains were washed 3 times for 10 min each in PBS/01.% Triton X-100 and then incubated overnight at 4 °C with 15 mg/ml BODIPY 493/503 (Molecular Probes, D-3922) diluted in the same buffer. The brains were washed 3 additional times with PBS/0.1% Triton X-100, mounted in Vectashield medium (AbCys) on a bridge slide, and stored at − 20 °C until imaged.

### Statistical analysis

Data are presented as the means ± standard deviation from three independent experiments unless noted. Statistical analyses were performed using R (R Core Team) and Prism software (GraphPad, San Diego, CA). The statistical tests applied are given in the figure legends.

## Supplementary information

Supplementary Information.

Supplementary Figure S1.

Supplementary Figure S2.

Supplementary Figure S3.

Supplementary Figure S4.

Supplementary Figure S5.

Supplementary Figure S6.
