# Supplementary material for: Spen modulates lipid droplet content in adult Drosophila glial cells and protects against paraquat toxicity
Source: Sci Rep. 2020 Nov 18;10:20023. doi: 10.1038/s41598-020-76891-9 (PMC7674452; doi:10.1038/s41598-020-76891-9)
Supplement: Supplementary file 2 — Supplementary Figure S1. [file 41598_2020_76891_MOESM2_ESM.pdf]

Girard et al, Supplemental Figure 1

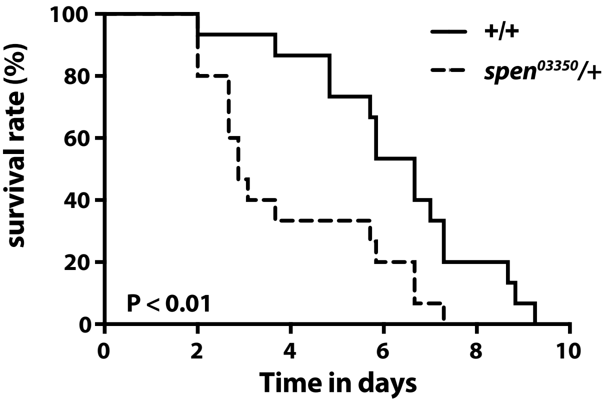

Figure S1. ***spen*** **heterozygous mutant flies are more sensitive to paraquat-induced lethality.** Survival rate of *spen* loss of function heterozygous mutants (*P{lacW}spen[03350]/+*) adult flies fed with paraquat (10 mM). *spen*^{03350} heterozygous flies are more sensitive to paraquat than wild-type flies (*w1118*). Log-rank Mantel-Cox test, P<0.0001.
