# Supplementary material for: Spen modulates lipid droplet content in adult Drosophila glial cells and protects against paraquat toxicity
Source: Sci Rep. 2020 Nov 18;10:20023. doi: 10.1038/s41598-020-76891-9 (PMC7674452; doi:10.1038/s41598-020-76891-9)
Supplement: Supplementary file 3 — Supplementary Figure S2. [file 41598_2020_76891_MOESM3_ESM.pdf]

Girard et al, Supplemental Figure 2

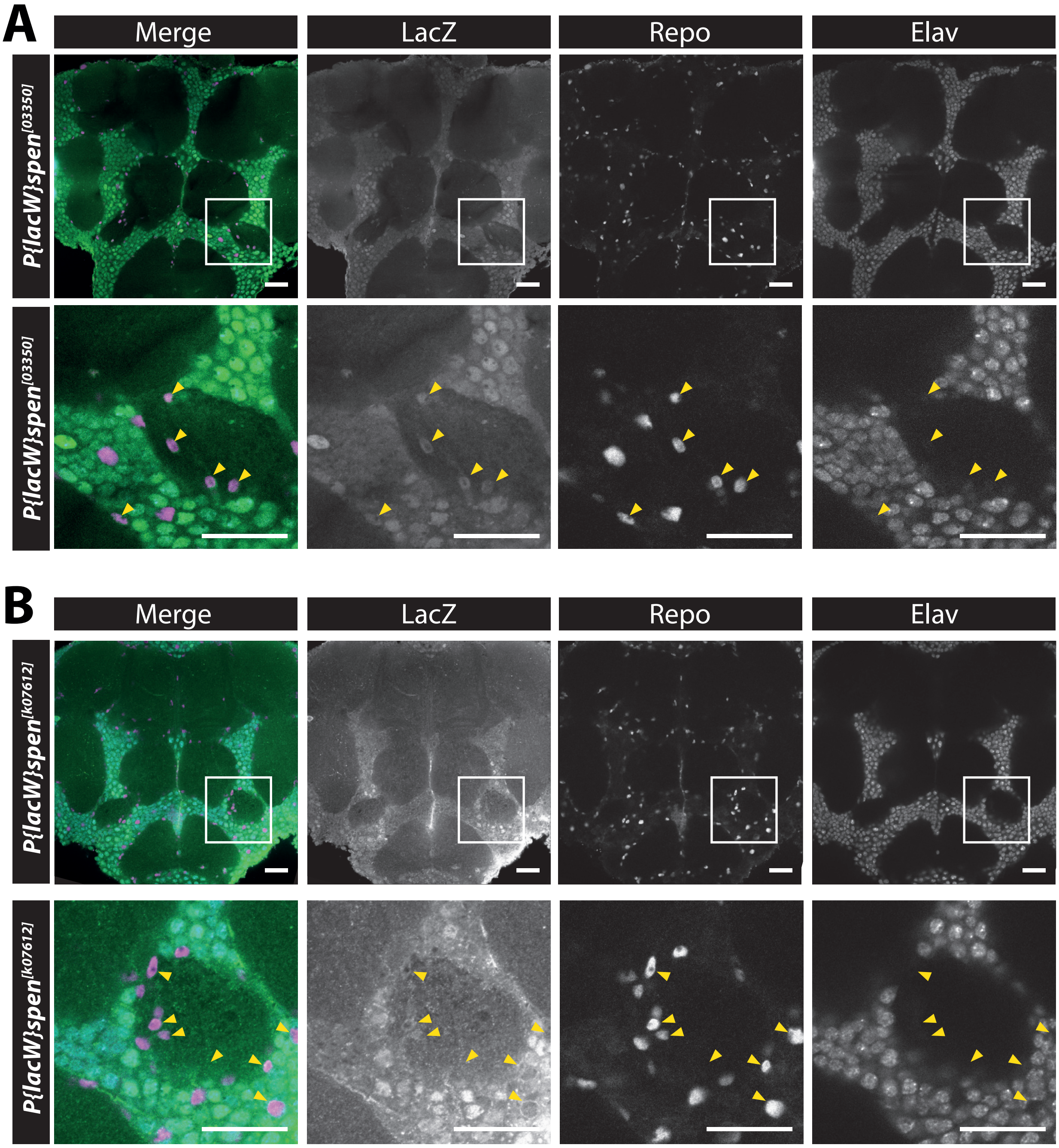

Figure S2. *spen* is expressed in both glia and neurons in *Drosophila* adult brain.
(A) *spen* expression domain in adult brain was assessed by immunostaining of LacZ (green) reporter gene in whole mount brain of 2 enhancer trap lines: *P{lacW}spen[03350]*, **(A)** and *P{lacW}spen[k07612]*, **(B)**. Glial cell nuclei are visualized using Repo staining (magenta) and neuron nuclei using Elav staining (cyan). Expression of *spen* in glial cells is indicated with yellow arrowheads. Scale bar: 25µm.
