# Supplementary material for: Spen modulates lipid droplet content in adult Drosophila glial cells and protects against paraquat toxicity
Source: Sci Rep. 2020 Nov 18;10:20023. doi: 10.1038/s41598-020-76891-9 (PMC7674452; doi:10.1038/s41598-020-76891-9)
Supplement: Supplementary file 4 — Supplementary Figure S3. [file 41598_2020_76891_MOESM4_ESM.pdf]

Girard et al, Supplemental Figure 3

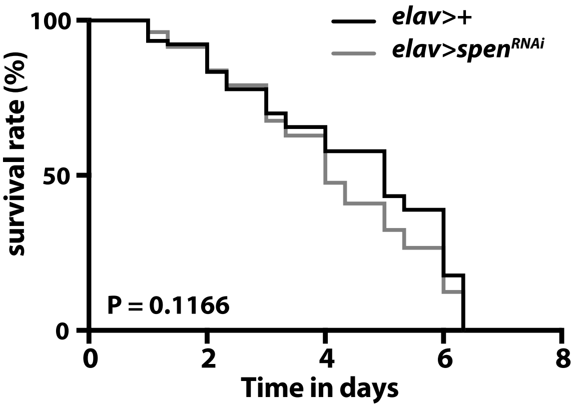

Figure S3. Knockdown of *spen* in neurons does not enhance the fly sensitivity to paraquat treatment.

Survival curves of adult male control flies (*elav-GAL4/+;;*) or flies with glial cell-specific spen knockdown (*elav-GAL4 ;UAS-spen^{RNAi} /+;*) fed with 20 mM paraquat. The curves represent the sum of three independent experiments with each N=20 flies per genotype. P=0.1166, not significant, by the log-rank Mantel–Cox test.
