# Supplementary material for: Spen modulates lipid droplet content in adult Drosophila glial cells and protects against paraquat toxicity
Source: Sci Rep. 2020 Nov 18;10:20023. doi: 10.1038/s41598-020-76891-9 (PMC7674452; doi:10.1038/s41598-020-76891-9)
Supplement: Supplementary file 5 — Supplementary Figure S4. [file 41598_2020_76891_MOESM5_ESM.pdf]

Girard et al, Supplemental Figure 4

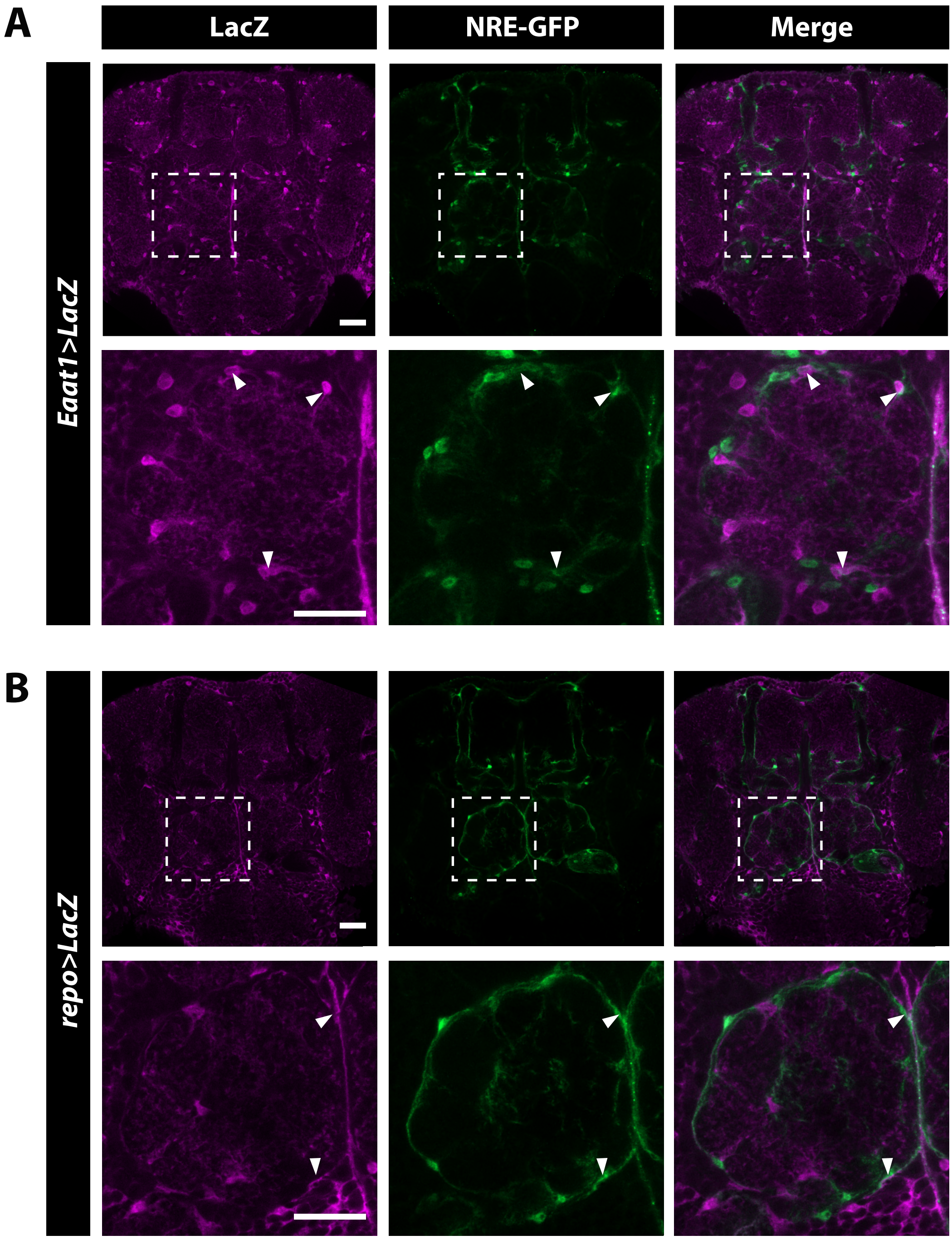

Figure S4. Notch signaling is activated in adult Eaat1+ glial cells .
Confocal microscopy of whole-mount brain of flies carrying Notch activation reporter NRE-GFP (green) expressing LacZ (magenta) under the control of glial driver *Eaat1-Gal4* (A) and *repo-Gal4* (B). NRE-GFP and LacZ co-localisation in glial cells Eaat1 or repo positive is indicated with white arrowheads. Scale bar: 25µm.
