# Supplementary material for: Spen modulates lipid droplet content in adult Drosophila glial cells and protects against paraquat toxicity
Source: Sci Rep. 2020 Nov 18;10:20023. doi: 10.1038/s41598-020-76891-9 (PMC7674452; doi:10.1038/s41598-020-76891-9)
Supplement: Supplementary file 6 — Supplementary Figure S5. [file 41598_2020_76891_MOESM6_ESM.pdf]

Girard et al, Supplemental Figure 5

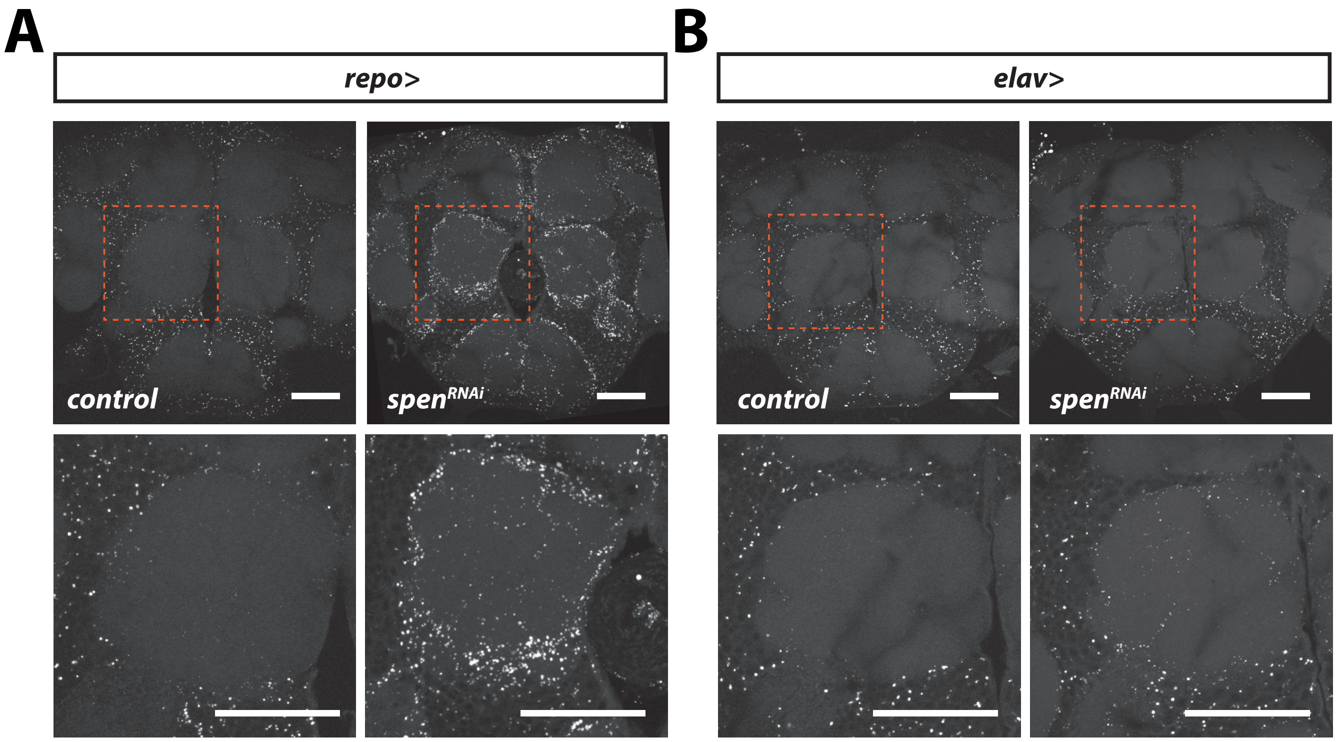

Figure S5. Lack of *spen* in glia but not in neurons promotes lipid droplet accumulation in adult *Drosophila* brain.

Lipid droplets labelled with BODIPY 493/503 (white dots) in whole-mount brain of flies expressing *spen*^{RNAi} specifically in glia (**A**, *repo-GAL4*) or neurons (**B**, *elav-GAL4*) compare to their respective control. As shown in the close-up, lipid droplets are accumulating in the neuropil area of the antennal lobe only flies lacking *spen* in glia but not in neurons. Scale bar: 25µm.
