# Supplementary material for: Spen modulates lipid droplet content in adult Drosophila glial cells and protects against paraquat toxicity
Source: Sci Rep. 2020 Nov 18;10:20023. doi: 10.1038/s41598-020-76891-9 (PMC7674452; doi:10.1038/s41598-020-76891-9)
Supplement: Supplementary file 7 — Supplementary Figure S6. [file 41598_2020_76891_MOESM7_ESM.pdf]

# Girard et al, Supplemental Figure 6

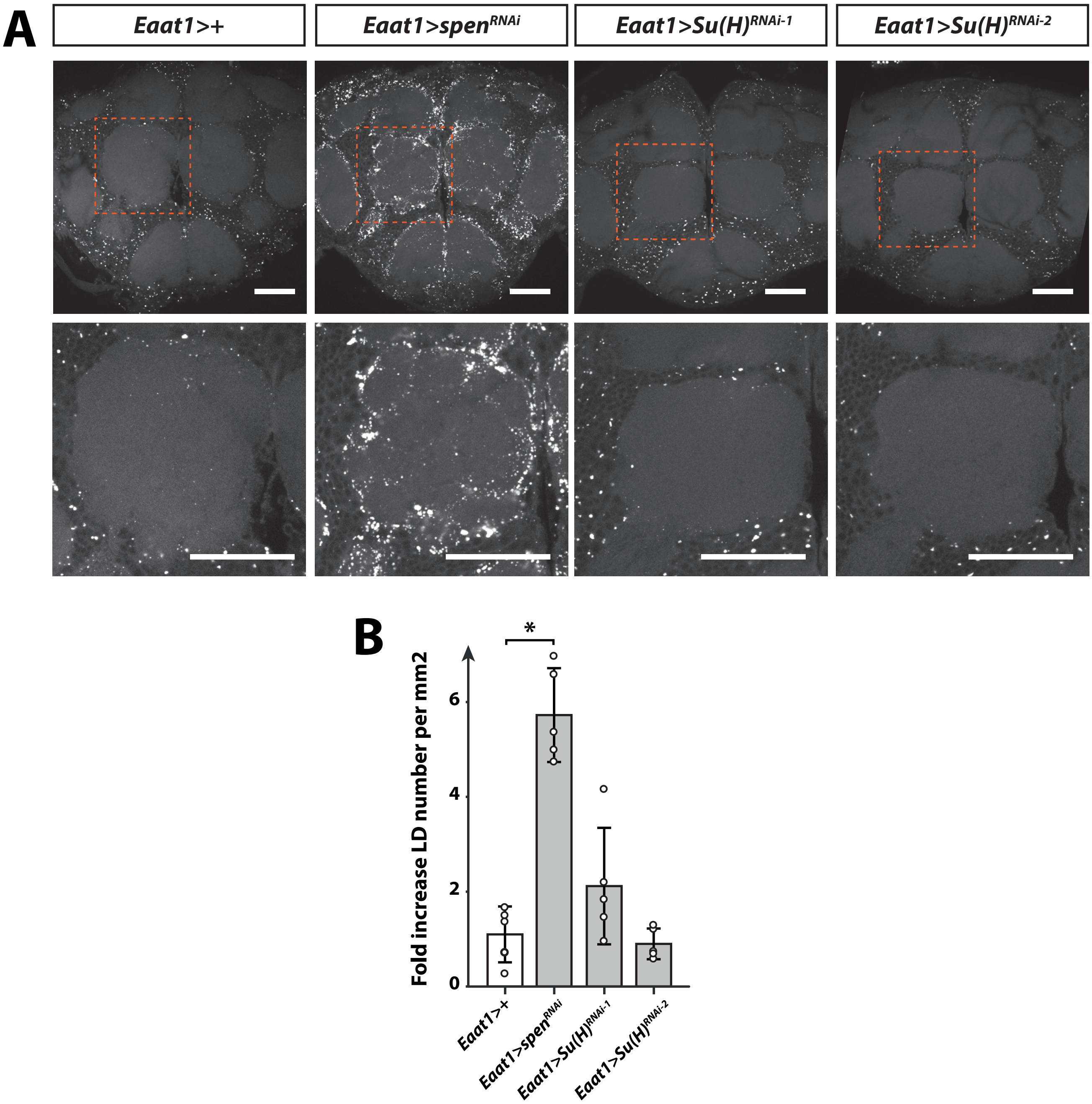


**Figure S6. *Su(H)* knockdown do not modify glial cell lipid droplet in adult *Drosophila* brain.** **(A)** Lipid droplets labelled with BODIPY 493/503 (white dots) in whole-mount brain of control flies *Eaat1>+* (*;Eaat1-GAL4/+;*) and flies expressing *spen*$^{RNAi}$ in glial cells *Eaat1>spen*$^{RNAi}$ (*;Eaat1-GAL4/UAS-spen*$^{RNAi;}$) was compared to flies expressing two different *Su(H)*$^{RNAi}$ (RNAi-1 : HMS05110 , RNAi-2: KK101550). As shown in the close-up, lipid droplets are accumulating in the neuropil area of the antennal lobe in *spen* knockdown but not in *Su(H)* knockdown. Scale bar: 25µm. **(B)** Quantification of BODIPY 493/503 labelled lipid droplets. Data are presented as the fold change in lipid droplets number (B) relative to control flies *Eaat1>+* (;Eaat1-GAL4/+;). n=5 brains per condition. Droplets were quantified using an automated ImageJ plugin described in the material and methods section. Non-parametric Kruskal-Wallis test, * P<0.05. Scale bar: 50µm.
